# Chemoenzymatic Epoxidation of Alkenes and Reusability Study of the Phenylacetic Acid

**DOI:** 10.1155/2014/756418

**Published:** 2014-01-27

**Authors:** Emilia Abdulmalek, Mahashanon Arumugam, Hanis Nabillah Mizan, Mohd. Basyaruddin Abdul Rahman, Mahiran Basri, Abu Bakar Salleh

**Affiliations:** ^1^Department of Chemistry, Faculty of Science, Universiti Putra Malaysia, 43400 Serdang, Selangor, Malaysia; ^2^Department of Biochemistry, Faculty of Biotechnology and Biomolecular Science, Universiti Putra Malaysia, 43400 Serdang, Selangor, Malaysia

## Abstract

Here, we focused on a simple enzymatic epoxidation of alkenes using lipase and phenylacetic acid. The immobilised *Candida antarctica* lipase B, Novozym 435 was used to catalyse the formation of peroxy acid instantly from hydrogen peroxide (H_2_O_2_) and phenylacetic acid. The peroxy phenylacetic acid generated was then utilised directly for *in situ* oxidation of alkenes. A variety of alkenes were oxidised with this system, resulting in 75–99% yield of the respective epoxides. On the other hand, the phenylacetic acid was recovered from the reaction media and reused for more epoxidation. Interestingly, the waste phenylacetic acid had the ability to be reused for epoxidation of the 1-nonene to 1-nonene oxide, giving an excellent yield of 90%.

## 1. Introduction

Epoxides are an important class of compounds in many industrial processes and often made by the epoxidation of alkenes [1]. These epoxides are valuable intermediates for laboratory syntheses as well as chemicals production as they can be easily transformed into a required functionality by means of regioselective ring opening reactions [2]. In addition, epoxides are also used as raw materials in many manufacturing industries, with some epoxides even exhibiting numerous biological activities [3, 4].

Many of these epoxides are synthesised using epoxidising agents such as metal catalysts or strong mineral acids; however, the yields of epoxide tend to be low and accompanied by side product formation as well as corrosion problems [5]. The synthesis of epoxides has gained more interest when enzymes began to be used as a catalyst with regards to the creation of environmentally friendly process [6]. The use of biological catalysts like lipases has its own advantage such as high regioselectivity, which can lead mainly to high purity in epoxide production [7, 8].

Prilezhaev epoxidation of alkenes with a peroxy acid is the most common method used in research laboratories and industries nowadays [9, 10]. Even though the Prilezhaev epoxidation protocol together with the lipase has been widely used for the manufacture of epoxides, the employment of peroxy acids is not a hygienic method because equivalent amount of acid waste is generated [11]. Therefore, special attention has to be given to the accumulation of unreacted peroxy acid, which could result in contamination of the end product and possible enzyme deactivation [11, 12].

Lately, we reported some of our findings on epoxidation of alkene using phenylacetic acid [13, 14]. The phenylacetic acid emerged as one of the effective perhydrolysis substrate, since a high stability of lipase was observed during the epoxidation process. As part of our ongoing research, we are interested in screening more variety of alkenes with phenylacetic acid as an oxygen carrier and Novozym 435—an immobilised *Candida antarctica* lipase B as a biocatalyst for the epoxidation process. Moreover, an examination was conducted to recover and reuse the excess phenylacetic acid from the reaction mixture in designing a more cheap, practical, safe, and environmentally friendly method to oxidise the alkenes.

## 2. Materials and Methods 

### 2.1. Materials

1-Nonene (98%), 1-heptene (97%), styrene (99%), cyclohexene (99%) and 1-methylcyclohexene (97%), cyclohexene oxide (standard), and chloroform-*d* were obtained from Sigma-Aldrich, USA. 1-Methylcyclohexene oxide (standard) was bought from Tokyo Chemical Industry, Japan. Chloroform, ethyl acetate, and toluene were purchased from Fisher Scientific, UK. Acetone, H_2_O_2_ (30%, w/w), and phenylacetic acid were purchased from Merck, Germany. A commercially known biocatalyst, CALB, immobilised on a macroporous acrylic resin (Novozym 435) was obtained from Novozymes Corporation, Denmark. The standards for 1-nonene oxide and 1-heptene oxide were prepared and identified as described by Rusch Gen Klaas and Warwel [15]. For GC-MS analysis, ethyl acetate and chloroform HPLC-grade were procured from Fisher Scientific, UK.

### 2.2. Experimental Procedures for Chemoenzymatic Epoxidation of Alkenes

In a typical experiment, epoxidation was carried out in a 50 mL capacity round bottom flask. Initially, alkene (1-nonene) (0.6 mmol) was evenly mixed in chloroform (10 mL) to form a constant solution. Neatly, phenylacetic acid (8.8 mmol) and Novozym 435 (1.7% wt/wt, 19.9 mg) were added to the mixture. The reaction was initiated by adding H_2_O_2_ (30% wt/wt, 4.4 mmol) in one step using an autotitrator (Metrohm, New Zealand). Ultimately, the reaction was carried out in a water bath shaker (Hotech Instrument, Taiwan) at temperature (35°C), time (12 h), and speed (250 rpm). For all experiments, the epoxide was synthesised at least in duplicate.

### 2.3. Sample Preparation for Quantitative Analysis

Samples were withdrawn at appropriate time intervals for a quantitative analysis. A 0.1 mL sample was diluted 100 times by mixing 9.9 mL of ethyl acetate (HPLC grade) and filtered using 0.45 *μ*M (Advantec, Japan) membrane filters before analysis. The yield was then determined by GC-MS (Agilent Technology, USA) and compared with an authentic standard prepared from the previous literature.

### 2.4. Quantitative Method

Analysis of epoxide yield was performed by using GC (Agilent Technology model 7890, GC system) coupled with a mass spectrometer, model 5975 C inert-MSD, with triple axis detector operated in the electron emission (EI) mode. The compound was separated on a (30.0 m × 0.25 mm) HP-5 ms column coated with 0.25 *μ*m film thickness of fused silica gel with 5% phenyl methylpolysiloxane.

### 2.5. Isolation and Purification of Phenylacetic Acid

The isolation of phenylacetic acid was performed using a 125 mL separating funnel. Initially, the reaction mixture was washed with distilled water to remove the remaining H_2_O_2_. After the phase separation, the organic layer was extracted out and dried over 5% (w/w) of Na_2_SO_3_ and Na_2_SO_4_, respectively. The crude mixture was purified by silica gel column chromatography with a mobile phase containing hexane and ethyl acetate (3 : 2). The purified combined fractions were evaporated under vacuum by rotary evaporator (Buchi, Switzerland). The phenylacetic acid was then characterised with GC-MS, ^1^H, and ^13^C NMR and compared with the reported data.

### 2.6. Spectroscopic Characterisation

Identification of final epoxide product was performed using spectroscopic analysis and matched with the reported data. The epoxide was periodically characterised by FT-IR (PerkinElmer-model 1650, USA) and GC-MS (Agilent Technology, USA) (see Supplementary Material available online at http://dx.doi.org/10.1155/2014/756418). The structure elucidation of epoxide was confirmed with ^1^H and ^13^C NMR (Jeol ECA, Japan). The optical rotation of the epoxide was determined by polarimeter (Jasco-model 2000, Japan).

## 3. Results and Discussion

### 3.1. Mechanism of Chemoenzymatic Perhydrolysis Reaction

**Scheme 1:**
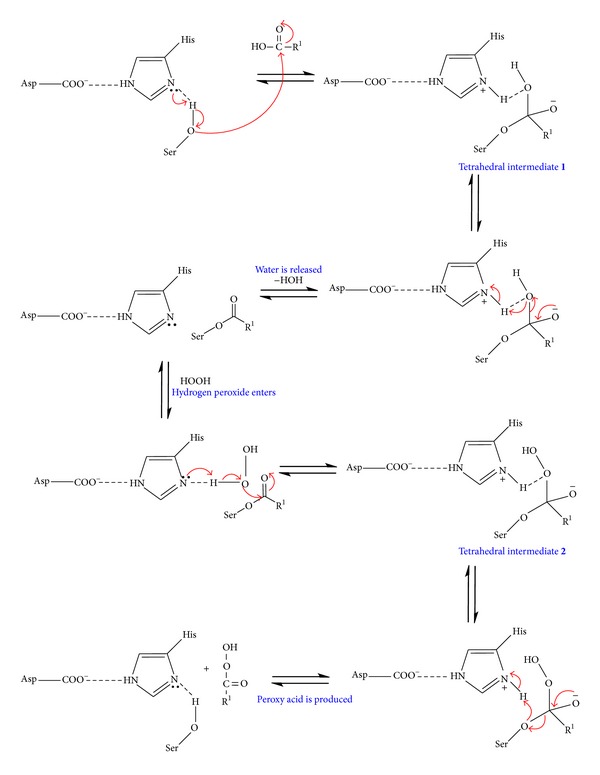
Mechanism of lipase-catalysed perhydrolysis reaction [17].

In the enzyme-catalysed epoxidation reaction, H_2_O_2_ acts as oxidising agent and converts the carboxylic acid into peroxy acid. In this study, the reaction was initiated using lipase Novozym 435 to catalyse the production of phenylacetic peroxy acid (the oxygen carrier) via the perhydrolysis of phenylacetic acid.  Scheme 1 describes the general reaction mechanism of lipase-catalysed perhydrolysis reaction.

The lipase catalytic site contains a catalytic triad composed of a histidine (His), serine (Ser), and an aspartate (Asp)/glutamic acid residue [16]. These three essential amino acids, located closely at the active site, play a major role in the cleaving ability of the lipase [17]. The serine amino acid has an OH group and serves as a nucleophile on the active site of lipase, thus forming a strong hydrogen bond between the “N” atoms in the imidazole ring of the histidine.

In the initial step, the lone pair electrons on the N atom of the histidine have the potential to accept the hydroxyl proton from the serine. This is due to a carboxyl residue of the aspartate/glutamic acid that forms a hydrogen bond with the imidazole ring of the histidine, making the N atom mentioned above be very electronegative. The serine residue makes a nucleophilic attack on a carbonyl carbon of the acyl group that belongs to the substrate (R^1^COOH) to form an intermediate known as tetrahedral intermediate **1**. The tetrahedral intermediate **1 **is stabilised by an oxyanion hole interaction, which is found in all lipases.

In the second step, the bond connecting carbon and oxygen atoms in the carboxylic acid is broken. The covalent electron on the oxygen thus moves to attack the hydrogen of the histidine and breaks the N–H linkage. The histidine group enhances the proton transfer from the hydroxyl group resulting in the release of a water molecule (HOH). When a H_2_O_2_ molecule comes, it attacks the carbonyl carbon of the serine complex resulting in the movement of the *π* electrons of the double bonds to oxygen, thus making it negative. Once again, a tetrahedral intermediate **2** is formed. In a final step, the bond between the serine and carbon of the acid moves to attack the proton of the histidine. As a result, the serine residue is ejected, and the product peroxy acid (R^1^COOOH) is released.

### 3.2. Mechanism of Chemoenzymatic Epoxidation Reaction

**Scheme 2:**
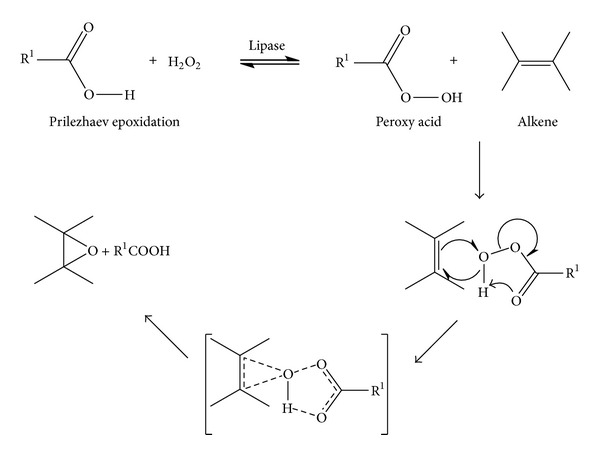
Mechanism of Prilezhaev epoxidation of alkene.

The *in situ* produced peroxy acid then oxidises the alkene, affording the respective epoxide and regenerating the carboxylic acid. The reaction mechanism for Prilezhaev epoxidation of alkenes is shown in Scheme 2.

### 3.3. Chemo Enzymatic Epoxidation of Alkenes

It is interesting to underline that all chemo enzymatic epoxidation reactions performed in the presence of phenylacetic acid gave good results, probably due to lipase stabilisation in mild phenylacetic acid. The summary of the epoxidation of different alkenes with Novozym 435, H_2_O_2_, and phenylacetic acid is shown in Table 1. Upon the oxidation of these alkenes via Prilezhaev epoxidation technique, epoxide yields of 75–99% were achieved with purities more than 90% in the gas chromatogram. Remarkably, the new developed system was efficient and convenient, since the epoxidation of the tested terminal alkenes was very fast in producing their respective epoxides with yields above 90%.

The yield of styrene oxide is lower than aliphatic epoxides, which was probably due to acid-catalysed hydrolysis and isomerisation of styrene oxide to diol and benzaldehyde, respectively. This phenomenon is similar to other lipase-catalysed epoxidation systems [18, 19]. In the case of 1-methylcyclohexene, incomplete yield was obtained within 12 h of reaction time. For this reason, the reaction time was prolonged to 16 h whereby higher yield of 1-methylcyclohexene oxide (90%) was achieved. Meanwhile, the epoxidation of cyclohexene with peroxy phenylacetic acid led to a moderate yield (75%) and selective cyclohexene oxide. The lower yield of cyclohexene oxide compared to other epoxide was because of the ring opening reaction of cyclohexene oxide to diol [8, 20].

It was worth mentioning that some authors formerly have reported that the lipase is not able at all to perform epoxidation reaction in one single addition of H_2_O_2_ [15]. However, this study demonstrated that chemo-enzymatic epoxidation was feasible with a single addition of H_2_O_2_, which provided an operational stability of lipase and thus promoted the lipase-mediated protocol in industrial application.

### 3.4. Recyclability of Phenylacetic Acid

**Figure 1:**
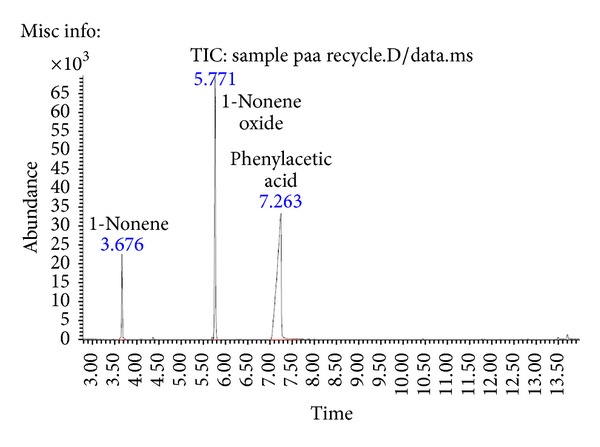
The GC chromatogram of epoxidation of 1-nonene using recycled phenylacetic acid.

Subsequent to epoxidation of various alkenes, recyclability of phenylacetic acid was studied in order to reduce the acid waste produced during the epoxidation reaction. In this study, phenylacetic acid was successfully isolated and purified from the reaction mixture and subjected to characterization with GC-MS and NMR. The GC chromatogram of epoxidation of 1-nonene using recycled phenylacetic acid is shown in Figure 1.

Only a small amount of recycled phenylacetic acid (0.3 g) was obtained after a series of isolation and purification steps. The result demonstrated that the highest yield of 90% 1-nonene oxide was achieved when phenylacetic acid was reused for the epoxidation reaction. This proved that phenylacetic acid is a very efficient perhydrolysis substrate as it can be reused for several epoxidation reactions.

In brief, the epoxidation reaction by lipase-mediated perhydrolysis of phenylacetic acid is a practical method, especially suitable for acid sensitive alkenes. The recyclability result exemplifies the importance of perhydrolysis substrate selection in chemo-enzymatic epoxidation of alkenes as this would control the cost of production and also promote the eco-friendly chemo-enzymatic epoxidation needs on both laboratory and industrial scales.

### 3.5. Comparative Study

Remarkably, the new developed system was efficient and convenient, since the epoxidation of the tested alkenes was rapid in producing the respective epoxides with yields above 90%. This implied the promise of practical application of this methodology. In particular, the epoxidation of styrene was found to be over 90% in 12 h as compared to other systems using dimethyl carbonate and ethyl acetate, which required at least 6 h dosing of H_2_O_2_ and additional 16 h of stirring time [15].

In addition, our method required 50% less H_2_O_2_ concentration and 20 h less time but produced almost 7% higher epoxide in comparison with the epoxidation of styrene in lactone [21]. On the other hand, our method also required 50% less H_2_O_2_ concentration and 70% less enzyme but gave 20% higher styrene oxide yield in comparison to Rusch Gen Klaas and Warwel [15] epoxidation that used dimethyl carbonate and ethyl acetate as perhydrolysis substrates. Furthermore, in comparison with the system performed in the urea-H_2_O_2_ and ethyl acetate [22], a 25% higher yield and much shorter reaction time were required for the epoxidation of styrene in our system with 10% less enzyme.

### 3.6. Spectroscopic Data

1-Nonene oxide: the analytical procedure was performed as reported by Abdulmalek et al. [13].

*1-Nonene Oxide*. Yield 97%; pale yellowish viscous oil. MS *m*/*z* (rel. int.): 142 [M^+^] (0.1), 113 (5), 99 (10), 85 (13), 81 (32), 71 (100), 69 (40), 68 (33), 67 (30), 58 (37), 57 (29), 56 (40), 55.1 (61); GC-MS *t*_*R*_ = 5.726 min; [∝]_*D*_^20^ = 0 (*c* 0.02, ethyl acetate).

*1-Heptene Oxide.* The standard for 1-heptene oxide was prepared according to a method reported by Rusch Gen Klaas and Warwel [15]. The inlet temperature was set at 260°C. The GC oven was initially maintained at temperature 40°C for 1 min; then it increased to 50°C at 1°C/min, was held for 12 min, and subsequently ramped to 260°C at 20°C/min. Identification of compounds was carried out with full SCAN mode (*m*/*z*) in the range of 40–160 amu. The SIM analysis was performed by observing ions at (*m*/*z* = 29.1, 41.1, 42.1, 55.1, 56.1) for 1-heptene and (*m*/*z* = 41.1, 55.1, 56.1, 71.1) for 1-heptene oxide, respectively.

*1-Heptene Oxide*. Yield 99%; colourless oil; IR *ν*_max⁡_ (cm^−1^): 2928, 1718, 1452, 1385, 1248, 1157, 981, 756; ^1^H NMR (500 MHz, CDCl_3_) *δ* 0.83 (3H, t, *J* = 7.0, CH_3_), 1.15–1.50 (8H, m, CH_2_-CH_2_), 2.41 (1H, dd, *J* = 2.8, 5.0, CHOCH_A_H_B_), 2.70 (1H, dd, *J* = 4.0, 5.0, CHOCH_A_H_B_), 2.83–2.87 (1H, br.m, CHOCH_2_) [23]; ^13^C-NMR (125.7 MHz, CDCl_3_) *δ* 14.0, 22.6, 25.6, 31.6, 32.4, 47.2, 52.5 [24]; MS *m*/*z* (rel. int): 114 [M^+^] (0.2), 113 (0.3), 85 (12), 71 (100), 67 (15), 58 (41), 56 (50), 55 (46), 43 (34), 42 (34), 41 (69); GC-MS *t*_*R*_ = 11.688 min; [∝]_*D*_^20^ = 0 (*c* 0.03, ethyl acetate).

*Styrene Oxide.* The inlet temperature was set at 280°C. The GC oven temperature was 50°C to 260°C at 15°C/min for 0 min. A full SCAN data (*m*/*z*) was performed in a mass range of 45–160. In SIM method, quantitation for styrene and styrene oxide was performed by monitoring the group of ions (*m*/*z* = 78.1, 103.1, 104.1) and (*m*/*z* = 89.1, 90.1, 91.1, 119.1), correspondingly. The purified product was then characterised with FT-IR, GC-MS, and ^1^H and ^13^C NMR.

*Styrene Oxide.* Yield 95%; colourless liquid; IR *ν*_max⁡_ (cm^−1^): 3039, 2990, 1606, 1476, 1254, 984, 873, 812; ^1^H-NMR (500 MHz, CDCl_3_) *δ* 2.81 (1H, dd, *J* = 2.6, 5.5, CHOCH_A_H_B_), 3.15 (1H, dd, *J* = 4.1, 5.5, CHOCH_A_H_B_), 3.87 (1H, dd, *J* = 2.6, 4.0, CHOCH_2_), 7.25–7.45 (5H, m, Ph); ^13^C-NMR (125.7 MHz, CDCl_3_) *δ* 51.2, 52.3, 125.5, 128.2, 128.5, 137.5; MS *m*/*z* (rel. int): 121 [M^+^ + 1] (3), 120 [M^+^] (32), 119 (43), 92 (30), 91 (100), 90 (47), 89 (57), 77 (6), 65 (15), 63 (16), 51 (10) [2]; GC-MS *t*_*R*_ = 5.475 min; [∝]_*D*_^20^ = 0 (*c* 0.10, ethyl acetate).

*Cyclohexene Oxide*. The inlet temperature was set at 280°C. Oven temperature programme was 50°C for 5 min and ramped to 160°C at 10°C/min. A full SCAN data (*m*/*z*) was performed in a mass range of 45–160. The SIM analysis was performed by observing ions at (*m*/*z* = 41.1, 53.1, 54.1, 67.1, 79.1, 81.1, 82.1) for cyclohexene and (*m*/*z* = 54.1, 55.1, 57.1, 69.1, 83.1) for cyclohexene oxide, respectively.

*Cyclohexene Oxide*. Yield 75%; colourless liquid; IR *ν*_max⁡_ (cm^−1^): 2926, 2855, 1363, 954, 883, 797; ^1^H-NMR (500 MHz, CDCl_3_) *δ* 1.15–1.32 (2H, m, CH_2_-CH_2_), 1.36–1.49 (2H, m, CH_2_-CH_2_), 1.75–1.87 (2H, m, CH_2_), 1.88–2.02 (2H, m, CH_2_), 3.11 (2H, m, CHOCH); ^13^C-NMR (125.7 MHz, CDCl_3_) *δ* 19.4, 24.5, 52.2 [22]; MS *m*/*z* (rel. int): 98 [M^+^] (4), 97 (14), 83 (100), 69 (31), 57 (30), 55 (40), 54 (43) [2], GC-MS *t*_*R*_ = 7.359 min; [∝]_*D*_^20^ = 0 (*c* 0.10, ethyl acetate).

*1-Methylcyclohexene Oxide.* The inlet temperature was at 280°C. Initial GC oven temperature was 50°C for 4 min and then ramped to 160°C at 10°C/min, and carrier gas flow rate-1 mL/min. A full SCAN data (*m*/*z*) was performed in a mass range of 45–160. For SIM Quantitative method: a group of ions was monitored in 1-methylcyclohexene (67.1, 68.1, 81.1, 96.2) and 1-methylcyclohexene oxide (55.1, 67.1, 69.1, 83.1, 97.1).

*1-Methylcyclohexene Oxide*. Colourless liquid; IR *ν*_max⁡_ (cm^−1^): 2927, 2861, 1450, 872, 835, 759; ^1^H-NMR (500 MHz, CDCl_3_) *δ* 1.10–1.32 (2H, m), 1.29 (3H, s, CH_3_), 1.34–1.48 (2H, m, CH_2_), 1.61–1.69 (1H, m, CH_2_), 1.78–1.94 (3H, m, CH_2_), 2.95 (1H, br.d, CH); ^13^C-NMR (125 MHz, CDCl_3_) *δ* 19.9, 20.3, 24.2, 24.9, 30.1, 57.8, 59.8 [22]; MS *m*/*z* (rel. int): 113 [M^+^ + 1] (1), 112 [M^+^] (13), 97 (100), 83 (53), 71 (20), 69 (30), 68 (20), 67 (25), 55 (72) [2]; GC-MS *t*_*R*_ = 6.480 min; [∝]_*D*_^20^ = 0 (*c* 0.10, ethyl acetate).

*Phenylacetic Acid.* For inlet, injector volume = 1 *μ*L, injector port temperature = 280°C, and splitless mode were used. The oven temperature was 50°C for 0 min; then it was increased to 260°C by 10°C/min. The epoxide was discovered by mass spectrometer detector operated at thermal aux temperature = 280°C with helium as a carrier gas, and flow rate = 0.9 mL/min.

*Phenylacetic Acid*. Pale yellow solid; ^1^H-NMR (500 MHz, CDCl_3_) *δ* 3.57 (2H, s, CH_2_), 7.17–7.29 (5H, m, Ph); ^13^C-NMR (125 MHz, CDCl_3_) *δ* 41.2, 127.5, 128.8, 129.5, 133.3, 178.1 [25]; MS *m*/*z* (rel. int): 137 [M^+^ + 1] (4), 136 [M^+^] (39), 92 (19), 91 (100), 65 (16); GC-MS *t*_*R*_ = 7.927 min.

## 4. Conclusion

Overall, the use of phenylacetic acid gave epoxide ranging from reasonable to good yields when compared to those reported when using strong carboxylic acids such as acetic acid and formic acid. Moreover, the recyclability result is very overwhelming, rendering this epoxidation method one of the best methods ever described. The method also revealed that only a mild operating temperature (35°C) and a milligram-scale of Novozym 435 (19.9 mg) were required to give higher catalytic activity towards the acid peroxidation process and limit the enzyme deactivation by H_2_O_2_. In this way, we found that the scope of the reaction can be extended particularly for industrial application.

## Supplementary Material

The mass spectrums of the respective epoxides are shown in the Supplementary material.

## Figures and Tables

**Table 1:** Novozym 435-mediated epoxidation of alkenes.

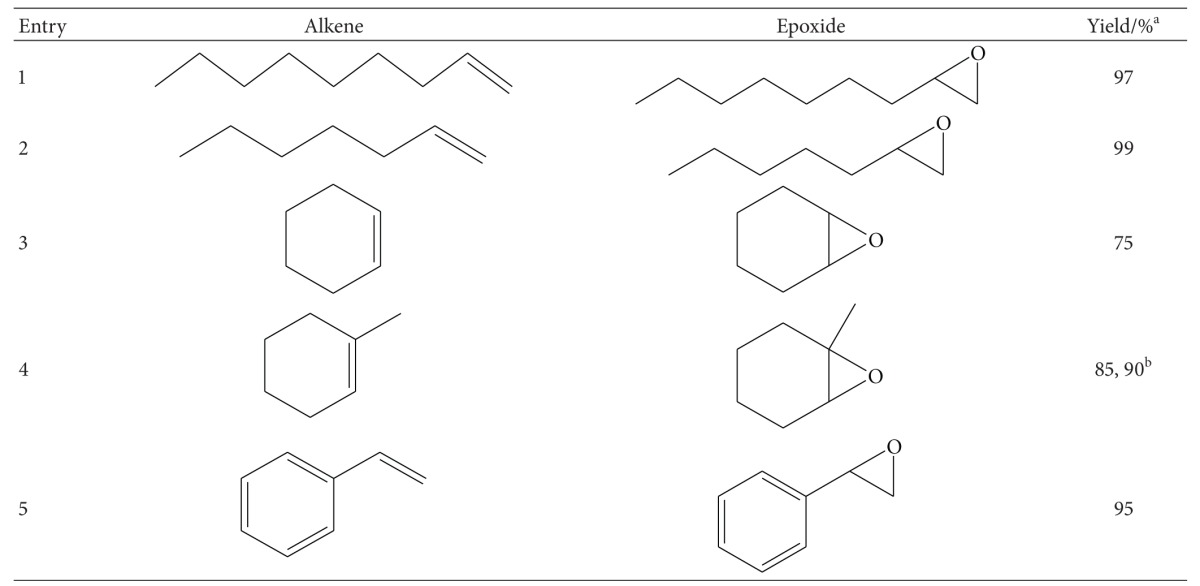

^a^Reaction was performed in 10 mL of chloroform at temperature (35°C) with alkene (0.6 mmol), phenylacetic acid (8.8 mmol), H_2_O_2_ (30% wt/wt, 4.4 mmol), Novozym 435 (1.7% wt/wt, 19.9 mg) and shaken at 250 rpm for 12 h. The yields were determined by GC-MS SIM method.

^b^The epoxidation yield was obtained after 16 h of reaction time.
