# Supplementary material for: Chemoenzymatic Epoxidation of Alkenes and Reusability Study of the Phenylacetic Acid
Source: ScientificWorldJournal. 2014 Jan 27;2014:756418. doi: 10.1155/2014/756418 (PMC3921943; doi:10.1155/2014/756418)
Supplement: Supplementary file 1 — The mass spectrums of the respective epoxides are shown in the Supplementary material. [file 756418.f1.doc]

Supplementary Data

GC-MS quantitative analysis of 1-nonene oxide

1-Nonene oxide

Mass spectrum of 1-nonene oxide


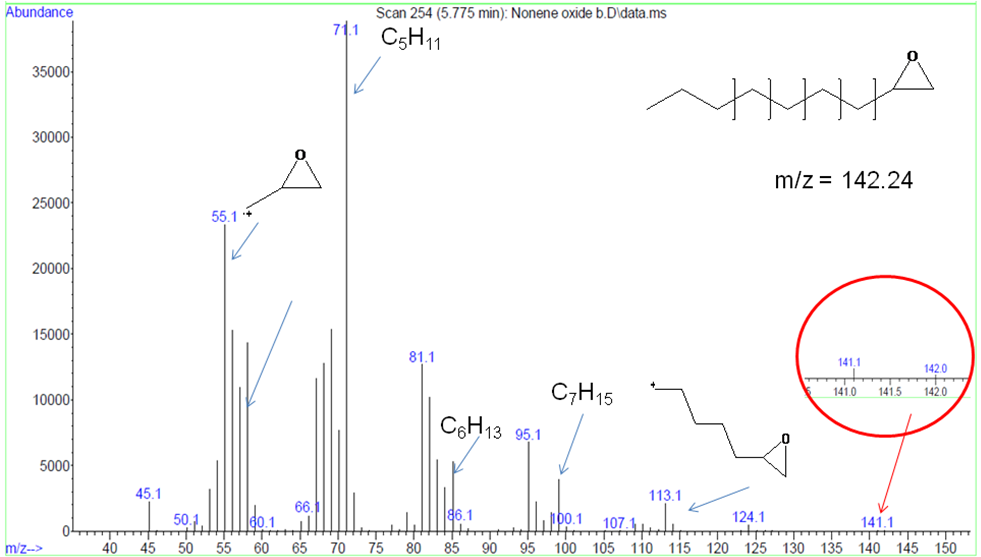


GC-MS qualitative analysis of 1-heptene oxide


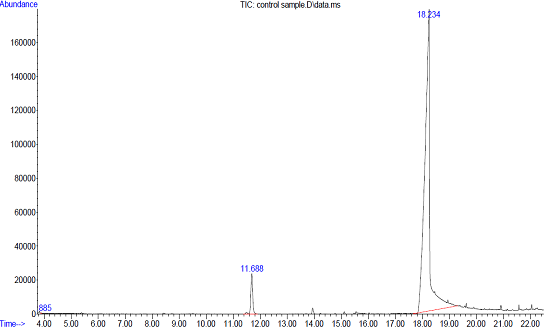


Phenylacetic acid

1-Heptene oxide

1-Heptene

GC-MS quantitative analysis of 1-heptene oxide

1-Heptene oxide

Mass spectrum of 1-heptene oxide

**
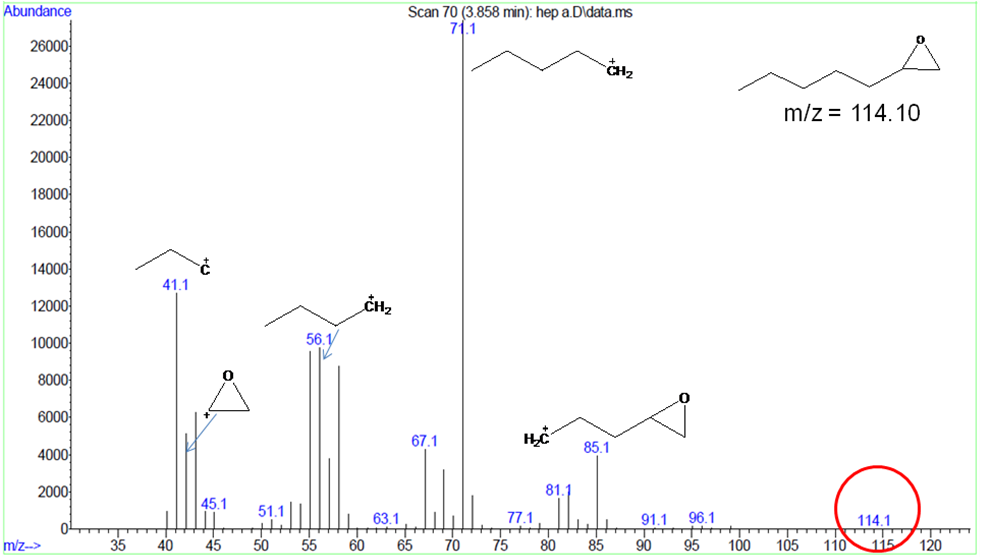
**

*m/z*

GC-MS qualitative analysis of styrene oxide


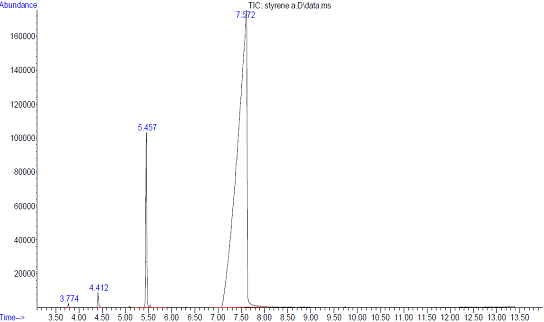


Phenylacetic acid

Styrene oxide

Styrene

GC-MS quantitative analysis of styrene oxide

**
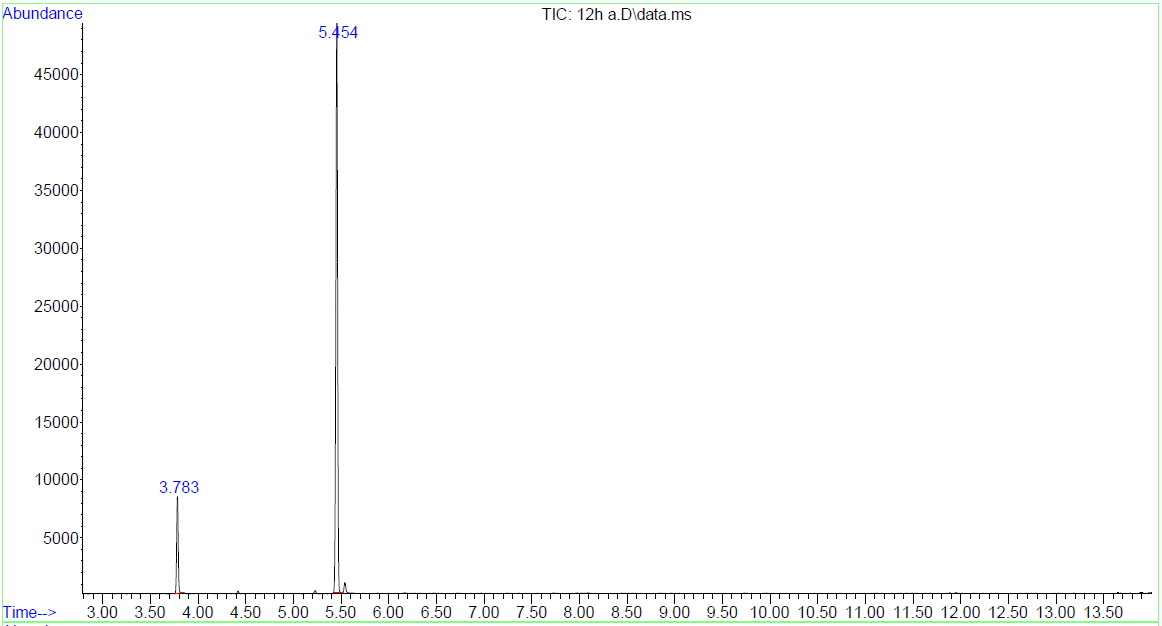
**

Styrene

Styrene oxide

Mass spectrum of styrene oxide

**
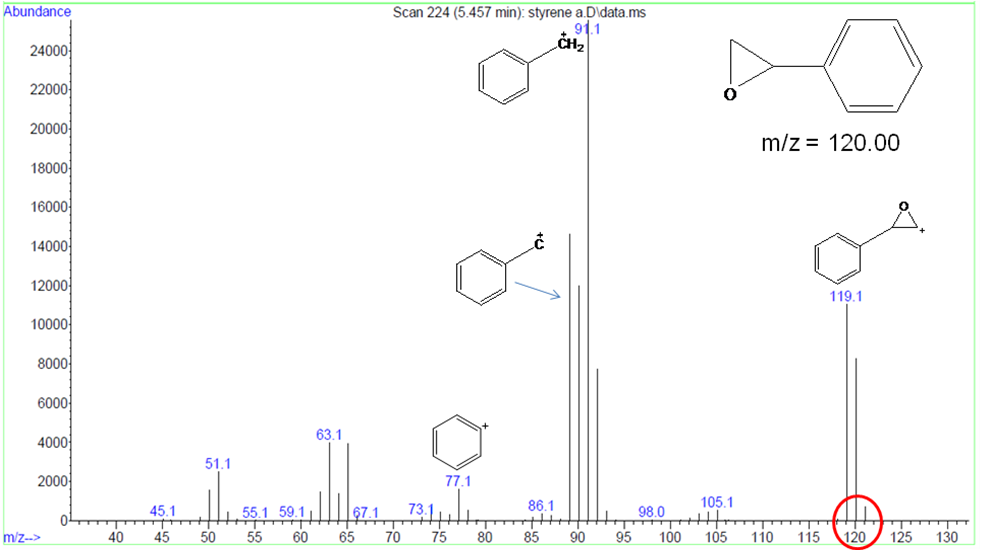
**

*m/z*

GC-MS qualitative analysis of cyclohexene oxide

Cyclohexene

Cyclohexene oxide

Phenylacetic acid

GC-MS quantitative analysis of cyclohexene oxide

Cyclohexene oxide

Mass spectrum of cyclohexene oxide

*m/z*

GC-MS qualitative analysis of 1-methylcyclohexene oxide.

1-Methylcyclohexene oxide

Phenylacetic acid

GC-MS quantitative analysis of 1-methylcyclohexene oxide

1-Methylcyclohexene oxide

Mass spectrum of 1-methylcyclohexene oxide

*m/z*
